# Codon usage signatures in Sabia and Chapare for host adaptation

**DOI:** 10.6026/97320630017891

**Published:** 2021-10-31

**Authors:** Himani Malhotra, Arvind Kumar

**Affiliations:** 1 Department of Biotechnology, School of Bioengineering and Biosciences, Lovely Professional University, Jalandhar Delhi G.T. Road, Phagwara, Punjab, INDIA -144411.; 2 Department of Biochemistry, School of Bioengineering and Biosciences, Lovely Professional University, Jalandhar Delhi G.T. Road, Phagwara, Punjab, INDIA -144411.

**Keywords:** Sabia virus, Chapare virus, Homo sapiens, codon usage, dinucleotides

## Abstract

Sabia and Chapare viruses in the Arenavirus family cause viral hemorrhagic fever among humans with a fatality rate of 30% with no treatment models. Therefore, it is of interest to document the codon usage, amino acid patterns and associated factors influencing the observed variations in Sabia and Chapare viruses for host adaptation. Multivariate statistical analysis revealed compositional constraint and host selection pressure influencing the viral codon usage patterns. These data suggests the codon usage signatures in Sabia and Chapare viruses for host adaptation in the human host implying its role in the rapid progression of the infection. Dinucleotides UpG and CpA were noted to be over-represented among the Sabia, Chapare viruses and human genomes. Strong restraint from the usage of CpG dinucleotides among viruses is linked with the molecular mimicry of the human immune system. Thus, the data reported from this study help in understanding the mechanism of viral adaptation inside the host genome for further consideration in drug discovery.

## Background:

Study of viruses to eradicate them globally is of great concern considering their highly detrimental association with all type of life forms including bacteria, archaea, and eukaryotes (human and agricultural sector, zoonotic threats) [1]. Arenaviridae represent family of viruses disseminating diseases among humans through direct or indirect contact with rodents [2]. Infections caused by Arenaviruses prevail more commonly in areas of South America, South Africa and have been described to be federated with severe disturbance among humans [3]. Arenaviridae mainly comprises of three genera Mammarenavirus, Reptarenavirus and Hartmanivirus including viruses infecting mammals, reptiles and fishes [4].The genome of Arenaviruses possessing negative sense single-stranded RNA encompasses two segments pertaining Small (S) RNA segment of size 3.4 kb encoding for envelope glycoprotein precursor (GPC) and the nucleoprotein (NP);Large (L segment) of size 7.2 kb encoding for matrix protein (Z) as well as the viral RNA-dependent RNA polymerase protein(L) [5]. On the basis of similarity in geographical distribution, antigenic properties and also on phylogenetic data genus Mammarenavirus have been subdivided into Old World Arenaviruse (OW) and New World Arenaviruse (NW)[7].Subgroups of OW and NW Arenaviruses include total 10 strains causing diseases among humans and are also examined as polyphyletic [6,7].Further New World Arenaviruse have been sub grouped into clades: A, B, C and D. Five viruses of clade B of NW Arenaviruse; known to be pathogenic among humans are Junin, Machupo, Guanarito, Sabia and Chapare [8]. Clade B viruses have been symbolized as an emergence among humans due to their categorization as type A pathogen and menace as a bioterrorism agent [9]. Sabia virus causing Brazilian haemorrhagic fever was first isolated in Sao Paulo in 1994 and Chapare virus causing Bolivian haemorrhagic fever was first isolated in Chapare Province in 2003 [10,11]. Yellow fever was the initial suspicion in case of the Sabia virus and also correlated with the Chapare virus infection as both had identified extensive liver necrosis [11]. The rodent host species for both the viruses are still unknown [10]. Apart from pervasion of studies for identification of therapeutic facilities for prevention and cure of Sabia and Chapare virus, no drug out of date being administered [4]. The availability of genomic sequencing data sprouted ample opportunities to study the riddles of the viruses at genomic level and to explore the convoluted methods showing that these viruses infect their host [12]. Therefore, study of synonymous codons that are considered to be equivalent and interchangeable has shown that alteration in synonymous codons affect the protein biogenesis which includes transcription, translation, posttranslational modifications, co translational modifications, hydrophobicity, hydrophilicity, the secondary structure of proteins, the abundance of tRNA and interaction between codon and anticodons [13-15]. Viral genomes, depends on the host machinery and cellular microenvironment for protein biogenesis, survival and progression of infection so this influences the requirement for exploration of viral host codon usage patterns [12,16]. Deciphering the variations and factors regulating the complicated patterns of codons and amino acids of viral genome may stimulate information regarding the regulation of host by viruses which may be utilized to design therapeutics and vaccines against virus with high accuracy [17]. Therefore, it is of interest to document the codon usage, amino acid patterns and associated factors influencing the observed variations in Sabia and Chapare viruses for host adaptation.

## Materials & Methods:

### Retrieval of Data:

Whole coding sequences of Sabia and Chapare viruses (Table 1 - see PDF) were downloaded from GenBank [18] and Virus Pathogen Resource database [19]. Coding sequence of H.sapiens (GRCh38.p13), common host to both the viruses was also extracted from GenBank [18] for further investigation (Figure 0 - see PDF).

### Assessment of parameters pertaining to Nucleotide composition and codon usage analysis:

Nucleotide composition properties like %A (Adenine),%G (Guanine), %C(Cytosine) and %T (Thymine); occurrence of GC (Guanine + Cytosine) at all the three positions of synonymous codons (GC1, GC2 and GC3); overall occurrence of AT and GC in Sabia and Chapare viruses were examined using CAIcal server [20]. RSCU (Relative synonymous codon usage) was computed by CodonW (Ver. 1.4.2) software [21].Codons having RSCU greater than 1.0 demonstrate positive codon usage biasness.

### Effective number of codons:

Effective number of codons (ENc) computed from CodonW [21] can have values from 20 to 61.Value equal to or close to 20 depicts that each amino acid has been encoded by one single codon only and there is no biasness whereas, value equal to or close to 61 shows that a particular amino acid can be encoded by more than one codon which is the case with no codon biasness. Codon usage patterns were computed by plotting ENc-GC3 plot [19].

### Neutrality plot:

Neutrality plot provides information about effect of mutational constraints and natural selection on genes of viral genome. Slope value of the regression line (close to or above 1) reflects the consequence of mutational constraint only, value (close to or below 0) reflects natural selection effect also [22].

### Correspondence analysis (CoA) of codon and amino acid usage data:

Correspondence analysis with a p-values less than or equal to 0.05 and 0.01 was performed using SPSS (Statistical Package for the Social Sciences) software to depict the changes in patterns of codon and amino acid in genome sequence [17,23].

### Estimation of Relative Dinucleotide Abundance:

Relative Dinuclotide Abundance (Pxy ) was analyzed using CAIcal server [37].Pxy value greater than 1.25 depicts over-representation of dinucleotides and Pxy value less than 0.78 show under-representation of dinucleotides [24].

### Computation of Codon Pair Score and Relative Synonymous Codon Pair Usage:

Relative Synonymous Codon Pair Usage (RSCPU) represented as ratio of observed frequencies to the expected frequencies of codon pairs. RSCPU values were computed by using an in-house BioPerl script and further RSCPU values are used to analyze the Codon pair Score (CPS) values for codon pairs of Sabia and Chapare viruses and its host human by using script. Positive CPS scores show over-representation of codon pairs, whereas, negative CPS scores depicts under-representation of codon pairs for virus and host [25].

### Codon adaptation index (CAI):

Values of CAI computed by CAIcal server ranges from 0 to 1 estimate the adaptation of viral genes inside the host cellular environment by using set of highly expressed reference genes. High CAI value (close to 1) of a concerned gene indicates immense level of similarity in its codon usage pattern with host and tremendous adaptation in host environment [17].

### Relative codon deoptimization index:

Relative codon deoptimization index (RCDI) analyzes the degree of acclimatization of viral genomes in host microcellular environment and were assessed by RCDI/eRCDI server [26].If RCDI value is low indicating better adaptation and increased translation of a viral gene segment in host system [27].

### Similarity index:

Similarity index estimates the magnitude of the impact of host genome in driving codon usage patterns of viruses. Similarity index values ranges from 0 to 1, value close to 1 implies a thorough effect of host on viral codon usage [28].

### Examination of tRNA adaptation index

tRNA adaptation index (tAI)estimates usage of tRNA by the coding sequences of viral genome. tAI defines adaptation level of coding sequence of virus with the corresponding tRNA pool of host cell by computing the presence of tRNAs for every codon of coding sequence [29].

## Results and Discussion:

Through assessment of RSCU data it was inspected that out of all possible codon sets (excluding start and stop codons) as shown in Table1 and 2(see PDF); 49.45% in Sabia and 47.45% in Chapare were preferred (RSCU greater than 1.0) codon sets respectively. Extensive analysis of genomic composition in the present study revealed that AU rich codons show preference over GC rich codons in Sabia and Chapare viruses shown in Table 3(see PDF). It was also perceptible from robust codon usage analysis as in Table 1(see PDF) and Table 2(see PDF) that Sabia and Chapare viruses had low codon usage biasness. Similar cases of RNA viruses showing low codon usage biasness have been reported earlier also [17,30-31]. Low codon usage biasness in viral genome reduces the competition of the virus with its host for usage of host machinery for synthesis and increases the efficiency of replication and easy adaptation inside the host cells [17,32].

Parameters affecting codon usage data were inferred from ENc versus GC3 plots and Neutrality plot [33]. If viral gene values prevail above or fall on the curve, mutational biasness is the only aspect affecting the codon usage. However, values lying below the curve signify the occurrence of natural selection also. In-depth study of the ENc versus GC3plot (Figure 1a and 1b - see PDF) of Sabia and Chapare viral nucleotide sequences revealed the clustering of viral genes below the ENc curve. Such an observation illustrated the integrated impact of mutational constraint and evolution on codon usage patterns of Sabia and Chapare genomes. Average ENc values were found to be 50.144 ± 2.07 for Sabia and 46.2375 ± 6.038 for Chapare virus. However, analysis of neutrality plot of Sabia and Chapare viruses revealed (Figure 2a and 2b - see PDF) 0.692, 0.821 slope of regression line signifying 69.2% and 82.1% impact of mutational pressure. Thus, it was evident that the effect of compositional constraint has been stronger than natural selection [33]. Further, Correspondence analysis was executed to classify the determinants causing variation in codon usage. Immense level of significant correlation of GC with Axis2 (one of the major axis of separation of genes) of RSCU data was observed in Sabia and Chapare viruses showing the influence of compositional constraint (Table 5 - see PDF).

RSCU data on Axis1 commence to show significant correlation with CAI, RCDI of Sabia and Chapare viral genomes (Table 5 - see PDF), thus, analyzing an indubitable affect of natural selection. Elements such as GRAVY (grand average of hydropathicity) and aromaticity show significant level of correlation with RSCU data on Axis2. Thus, codon usage patterns of the Sabia and Chapare viruses found to be a complex interplay of diverse crucial determinants. This analysis predicts that codon usage patterns of both Sabia and Chapare viruses found to be afflicted by many factors like mutational biasness, natural selection; hydropathicity and aromaticity [34,35].Yet, in spite of a convoluted interplay of various determinants, compositional constraint was found to play the most dominant role in shaping codon usage of Sabia and Chapare viruses.

Further vigorous analysis of relative dinucleotide abundance in Sabia and Chapare viruses revealed that UpG and CpA dinucleotides were over-represented and dinucleotide CpGs, were found to be under-represented among Sabia and Chapare viral genome (Figure3 (a, b) - see PDF). Similar patterns of dinucleotides were also observed to be highly preferred in H. sapiens also. Dinucleotides have a great influence on codon usage pattern and such feature of under-representation of CpGs dinucleotide has been observed in various genomes of RNA viruses [36]. It has been proposed that coding sequences of viral pathogens having unmethylated CpG have been recognized as pathogen signature's by host receptor Toll like receptor 9 (TLR9) and stimulates innate immune responses in host(human)[37]. However, presence of under-representation of CpGs dinucleotide will decline the host immune response and bring about increase in viral infection among host. Also, analysis of viral genome data in our study proved that selective pressure with evolution has influenced the dinucleotide pattern and also codon usage of humans.

Extensive analysis of Sabia virus codon pairs reported that based on RSCPU values; 1237 out of total 3721 codon pairs (excluding stop:stop and stop:sense codon pairs)were found to be over-represented and 519 were under-represented. GCG-ACC codon pair coding for Alanine-Threonine was utmost over-represented and codon pair ACA-AAG coding for Threonine-Lysine as shown in Table 1(see PDF) was utmost over-represented. Interestingly, in Sabia virus where 55.5% matched with the over-represented codon pairs in H. sapiens. Similar trend was also evident among under-represented codon pairs where 48.16% matched with that of the under-represented codon pairs of the human genome. Similarly, thorough study of RSCPU values of Chapare virus explained that 1249 out of 3721 were found to be over-represented, 533 were under-represented. CGG-CCC codon pair coding for Arginine-Proline was utmost over-represented and codon pair UUC-GAG, encoding for Phenylalanine-Glutamate pair as in Table 2,was examined as utmost under-represented in Chapare virus. Interestingly, in Chapare virus 56.6% matched with the over-represented codon pairs and 47.65% matched with that of the under-represented codon pairs of the human genome.

Similar trend was also evident among under-represented viral codon pairs as 254 out of 533 (Dinucleotide pattern NNU-GNN (UpG dinucleotide) was depicted as one of the most prevalent (10.6% in Sabia virus and 11.04% in Chapare virus) as compare to the other over-represented codon pairs (Figure 4a, b - see PDF). In addition, methodical inspection at the codon pair interface (cP3-cA1) determined that UpG, CpA, and CpU dinucleotides, were prevalent at the codon-codon junctions in Sabia and Chapare viruses (Figures 4 a, b - see PDF). Interestingly, exactly same dinucleotide patterns were also noted to be predominant among the codon pairs in H. sapiens, revealing efficient adaptation of viruses in humans.

Sabia and Chapare viruses were found to display antagonism with human host (Table1 and 2 - see PDF). Past study revealed that antagonistic codon patterns decreases the translational efficacy but leads to proper and correct folding of viral proteins. Various parameters such as Codon adaptation index, Relative codon deoptimization index and Similarity index of viral genes analyzed the adaptation of viruses among host Homo sapiens. The average value of Codon adaptation index of Sabia virus was 0.76±0.03 and Chapare virus was 0.75±0.02.The average RCDI value of Sabia virus was 1.40±0.04 and Chapare virus was 1.41±0.23. The SiD values computed for the Sabia virus and Chapare virus was 0.072 and 0.073 showing the low impact of human host on viral codon biasness. These results predict high level of adaptation of viruses in H.sapiens [17,25]. Examination of highly favoured codons in Sabia, Chapare viruses and isoacceptor tRNAs present in human cells divulged that 9 codons out of 18 (Table 5 - see PDF) highly favoured codons in Sabia virus;10 out of 18 in Chapare virus (Table 6 -see PDF) correspond together with the relevant isoacceptor tRNAs present in human hosts. On the whole the highly preferred codons examined in viral coding sequences utilize suboptimal isoacceptor tRNAs present in human cells (Table 5 and 6 - see PDF). Similar results have also been reported for Nipah virus to recognize the usage of suboptimal tRNA isotype. It has been proposed that throughout the initial phase of an infection; the utilization of suboptimal isoacceptor host tRNAs might lead to gradual and exact translation of viral proteins [38].

## Conclusion:

We report the codon usage patterns of Sabia and Chapare viruses relative to the host codon usage pattern. Data shows a weak codon bias in Sabia and Chapare viruses to help in adaptation to the host. Mutation is affecting variation in codon patterns of viral sequences than hydropathicity and aromaticity. Thus, the data reported from this study help in understanding the mechanism of viral adaptation inside the host genome for further consideration in drug discovery.
